# Cobalamin and Folate Status among Breastfed Infants in Bhaktapur, Nepal

**DOI:** 10.3390/nu10050639

**Published:** 2018-05-18

**Authors:** Ram K. Chandyo, Manjeswori Ulak, Ingrid Kvestad, Mari Hysing, Merina Shrestha, Suman Ranjitkar, Arve Ulvik, Per Magne Ueland, Laxman Shrestha, Tor A. Strand

**Affiliations:** 1 Centre for Intervention Science in Maternal and Child Health, Centre for International Health, University of Bergen, P.O. Box 7800, 5020 Bergen, Norway; tor.strand@uib.no; 2 Department of Community Medicine, Kathmandu Medical College, P.O. Box 21266, Sinamangal, Kathmandu, Nepal; 3 Department of Child Health, Institute of Medicine, P.O. Box 1524, Kathmandu, Nepal; manjeswori@gmail.com (M.U.); drmerinashrestha@gmail.com (M.S.); sumanra7@gmail.com (S.R.); laxmanshree12@gmail.com (L.S.); 4 Regional Center for Child and Youth Mental Health and Child Welfare, West, Uni Research Health, P.O. Box 7810, 5020 Bergen, Norway; ingrid.Kvestad@uni.no (I.K.); mari.hysing@uni.no (M.H.); 5 Department of Clinical Science, University of Bergen, P.O. Box 7800, 5020 Bergen, Norway; arve.ulvik@uib.no (A.U.); per.ueland@ikb.uib.no (P.M.U.); 6 Innlandet Hospital Trust, P.O. Box 990, 2629 Lillehammer, Norway; 7 Lillehammer University College, P.O. Box 952, 2604 Lillehammer, Norway

**Keywords:** cobalamin, folate, infants, combined B_12_, methylmalonic acid, homocysteine and Nepal

## Abstract

Cobalamin and folate are crucial micronutrients during infancy and they are required for growth and cognitive development. Due to the monotonous and predominantly vegetarian-based complementary feeding and poor maternal micronutrient status, infants from low- and middle-income countries are susceptible to cobalamin deficiency. However, data on plasma cobalamin and folate and the functional markers methylmalonic acid and total homocysteine from breastfed infants in Nepal are still needed. We collected plasma samples from 316 6–11-month-old breastfed infants with a length-for-age of less than minus one *z*-score and analyzed blood for plasma folate, cobalamin, methylmalonic acid and total homocysteine concentrations. Cobalamin deficiency (plasma cobalamin <148 pmol/L) was found among 11%, whereas 24% of the infants had plasma cobalamin concentrations between 148–221 pmol/L. Elevated total homocysteine (>10 µmol/L) and methylmalonic acid (>0.28 µmol/L) indicating functional cobalamin deficiency were found among 53% and 75% of the infants, respectively. Based on a combined indicator of cobalamin status, 58% were found to have low cobalamin status. However, folate deficiency (<10 nmol/L) was not found as the lowest value of plasma folate was 20.7 nmol/L. It is important to examine the extent to which poor cobalamin status during infancy has immediate or long-term consequences.

## 1. Introduction

Both cobalamin (vitamin B_12_) and folate are important for proper growth and cognitive development as they are required for normal cell division, maturation of red blood cells and myelination of the central nervous system [1,2,3]. Due to the monotonous vegetarian-based complementary feeding practices and/or maternal malnutrition in low- and middle-income countries, cobalamin deficiency is common [4,5,6,7,8]. Deficiencies of cobalamin and folate during the early stages of life may not only lead to immediate negative consequences for growth and increased morbidity, but have also been associated with later neurodevelopment [9,10,11,12,13]. However, there are no specific clinical manifestations that can be used to diagnose cobalamin and folate deficiencies during the early stages, particularly among infants. The available biochemical indicators of cobalamin and folate status are of different sensitivity and specificity [14,15]. Moreover, only 6–20% of plasma cobalamin, which is widely used to define cobalamin deficiency [16], exists in its active form, bound to transcobalamin [17]. This underlines the importance of the functional markers methylmalonic acid (MMA) and total homocysteine (tHcy), and a recently proposed combined indicator of cobalamin status (3cB_12_) [18].

For infants of 7–12 months, 0.7 μg of cobalamin per day is set as the requirement, but achieving this amount from vegetarian-based complementary feeding is unlikely [19,20]. Breast milk is an excellent source for many vitamins and minerals, but not for cobalamin, and hence many exclusively breastfed infants are at risk for cobalamin deficiency [21]. Infant cobalamin status also depends on maternal status during pregnancy [22,23,24,25]. Infants born to mothers with low cobalamin status are more likely to be cobalamin-deficient compared to infants of mothers with normal status, probably due to poor trans-placental transfer and low storage of cobalamin [14,26].

Poor cobalamin status in combination with high serum folate levels may exacerbate neurological damage and could also worsen poor cognitive function in adults [27]. In the one-carbon metabolic pathway, poor function of the cobalamin-dependent enzyme methionine synthase may lead to trapping of folate as 5-methyl tetrahydrofolate [28]. Deficiencies of both cobalamin or folate also may cause megaloblastic anemia leading failure to thrive, and developmental delay [29].

In Nepal, stunting is a common form of malnutrition among children affecting approximately one third of children under the age of 5. Of women, 17% are undernourished (BMI < 18.5 kg/m^2^) (NDHS 2016). The prevalence of anemia is also high affecting approximately 53% [30]. However, data on status of micronutrients including cobalamin and folate from breastfed infants from Nepal are still lacking. Because of rapid growth and development, infants are particularly vulnerable to micronutrient deficiencies [6,31]. A focus on maximizing nutrition during the second half of infancy is crucial as most of the growth faltering starts during this period [32,33], which may also have long-term and intergenerational consequences [34,35]. In the present study, we analyzed cobalamin and folate status, and explored its determinants among infants 6–11 months residing in the Bhaktapur municipality and peri-urban communities of Nepal.

## 2. Methods

### 2.1. Study Site and Population

Bhaktapur municipality is located ~15 km east of Kathmandu, the capital city of Nepal. As per the last census it has a total population of 81,748 mostly belonging to the Newar ethnic groups and residing in 17,639 households, with 5053 children below five years of age. After the devastating earthquake on 25 April 2015, most of the households were damaged and most of the residents shifted to peri-urban areas of the municipality. Although most of the families are traditionally engaged in agriculture, other main incomes are from small-scale self-business, daily wage working and other labor. Ownership of land and houses are social indicators as most of the families that have migrated from other parts of Nepal, usually live in rented dwellings. Piped government supplies of drinking water to households or courtyards are common but supplies are limited to one hour per day or even fewer.

### 2.2. Methods and Study Design

We collected baseline blood samples from infants participating in a community-based, randomized, double blind clinical trial evaluating efficacy of daily cobalamin supplementation for one year on growth and cognitive development. A total of 600 children 6–11 months old with a length-for-age of less than minus one *z*-score were enrolled in the study. For the present study, results of the blood analyses are available only from the first 316 enrolled infants. Details of the study design and main outcomes of the study have been published elsewhere [36]. Apart from socio-economic and breastfeeding information, we also collected details on the use of micronutrient supplementation from the mothers during pregnancy. Infants with acute illness, severe systemic illness requiring hospitalization, severe malnutrition or taking B vitamins at the time of enrolment were not included in study.

### 2.3. Ethics

The main study has obtained approval from the Nepal Health Research Council (NHRC, #233/2014) in Nepal and from the Regional Committee for Medical and Health Research Ethics (REC #2014/1528) in Norway and registered at clinicaltrials.gov (NCT02272842). Written informed consent from one of the parents (usually mother) was obtained. For illiterate parents, we took thumbprints in the presence of an impartial witness. In case of anemia (defined as hemoglobin <11 g/dL), iron supplementation was given according to national guidelines. Implementation of the study was as stated in the latest version of the Helsinki Declaration.

### 2.4. Blood Sampling and Biochemical Analyses

Most blood samples (80%) were taken at the study clinic after 12 noon, and collected from one of the cubital veins into heparinized polypropylene tubes (Sarstedt, Germany) which were protected from direct sunlight exposure. A total of 24% of the infants did not have any meal or snacks, during the day before blood sampling, whereas only 2% were not breastfed. The hemoglobin concentration was analyzed immediately following blood sampling with Hemocue (Ångelholm, Sweden), which was calibrated as per the guidelines defined by the manufacturer. The heparinized blood was centrifuged for 10 min at 700 g within 10 min after venipuncture and kept at −196 °C liquid nitrogen located at the field site clinic. Plasma was separated, transferred into polypropylene vials (Eppendorf, Germany) and stored at −80 °C in Nepal until it was transferred to Norway on dry ice. Plasma tHcy and MMA were analyzed by gas chromatography-mass spectrometry (GC-MS) based on methylchloroformate derivatization [37]. The plasma concentrations of folate and cobalamin were determined using microbiological assays [38,39] based on a colistinsulfate-resistant strain of *Lactobacillus leichmannii*. The assay has been adapted to a microtiter plate format and is carried out by a robotic workstation. The between-day coefficient of variation ranged from 2–8% for MMA and tHcy and was 5% for both cobalamin and folate. All biochemical analyses were done at Bevital Laboratory, Bergen, Norway (www.bevital.no).

### 2.5. Definitions

The cut-off values for altitude adjusted (1400 m) anemia, low cobalamin, low folate, high MMA and high tHcy are as per the WHO/CDC guidelines: 11.3 g/dL, <148 pmol/L, <10 nmol/L, >0.28 μmol/L, and >10 µmol/L, respectively [15,40]. Functional cobalamin deficiency is considered when MMA or tHcy are elevated. The combined indicator of cobalamin (3cB_12_) is calculated based on the three biomarkers (cobalamin, MMA and tHcy) as suggested by Fedosov et al. [18]. A score below <−0.5 is referred to as low cobalamin status. Underweight, stunting and wasting are defined as weight for age, length for age and weight for length *z*-scores below −2 *z*-score, respectively, as compared with WHO growth chart [41]. Breastfeeding patterns are categorized as exclusive, predominant, partial or none as per the definitions of Labbok and Krasovec and information were collected at the time of enrolment [42].

### 2.6. Statistical Analyses

Descriptive statistics such as means, medians, percentiles, interquartile ranges, standard deviations and 95% confidence intervals (CI) were calculated for baseline characteristics and for plasma concentrations of cobalamin, folate, MMA, tHcy, and Hb, as well as the combined indicator 3cB_12_. To identify determinants for cobalamin and biomarker status, we used the 3cB_12_ and log-transformed tHcy, MMA and cobalamin concentrations as the dependent variables in multiple linear regression models. Candidate variables for these regression models are those listed in Table 1. We selected the variables in a purposeful manual selection procedure as described elsewhere [43]. In short, all variables were assessed in crude models, variables that were significant at a 0.2 level were included in a more saturated model. The candidate variables that were not significant in the crude model were then included in this multiple model one at a time. If the variables became significant, they were retained in the model. After the final step of the variable selection process, only variables that were significant on the 0.05 level were kept in the model. We decided *a priori* to include age of the child (in months) in all regression models. Statistical analyses were undertaken using Stata^®^, version 15 (STATA Corp, Houston, TX, USA).

## 3. Results

### 3.1. General Characteristics and Breast and Complementary Feeding Practices

The mean age of the 316 enrolled infants was 8.3 months and 53% were male. One in every second family lived in a rented house and 50% were living in joint families. Most of the children were delivered at health centers (95%) with normal vaginal delivery (74%). At birth, 17% of the children had low birth weight (birth weight <2500 g) (Table 1). Almost all mothers (96%) had regular antenatal check-ups and took iron supplementation which usually also contained folic acid. Calcium supplementation during pregnancy is also universal practice in our setting and usually starts from the second trimester.

Most of the infants (85%) received breast milk within 24 h of delivery (56% within one hour), but the prevalence of exclusive breastfeeding at 3 and 6 months was only 43% and 10%, respectively. Early introduction of homemade cereals (lito) or Cerelac (Nestlé) was a common practice as 40% of infants were given these foods by 3 months of age but this is recommended only after 6 months of age. An Ayurvedic liquid (*Janamghuti*) was also given to 54% of infants, often within 3 months after delivery. One third of the infants were stunted (<−2 *z*-score), whereas the prevalence of underweight infants was 19% (Table 1).

### 3.2. Plasma Cobalamin, Folate, MMA, tHcy and 3cB_12_

**Figure 1:**
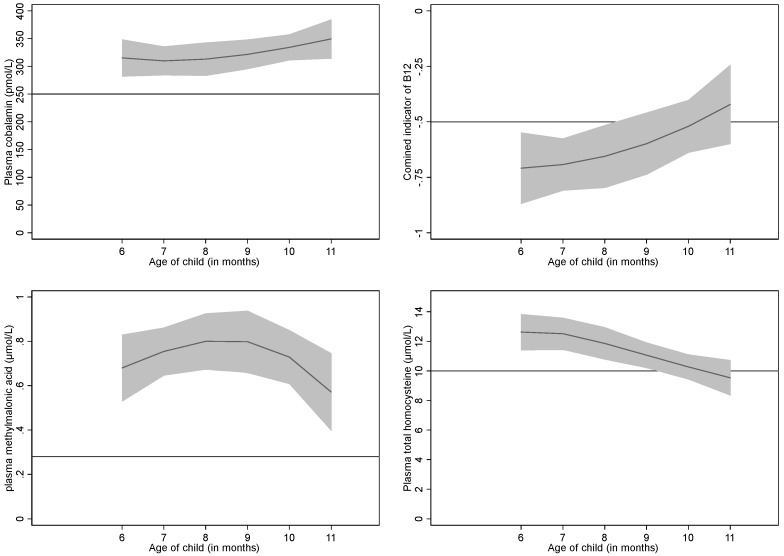
Concentrations of plasma cobalamin, combined indicator (3cB_12_), methylmalonic acid and total homocysteine by age among 316 breastfed infants in Bhaktapur, Nepal. The *Y* axis is the concentration of the biomarker and the *X* axis is the age of the infant (month). The shaded area represents the 95% CI of the depicted association. The horizontal lines indicate commonly used cut-offs for the different biomarkers (250 pmol/L for cobalamin, −0.5 for 3cB_12_, 0.28 µmol/L for MMA, and 10 µmol/L for tHcy).

The geometric mean (95% CI) concentration of cobalamin, folate, tHcy and MMA were 271.6 (257.8–286.2) pmol/L, 61 (59–64) nmol/L, 10.8 (10.3–11.4) µmol/L, and 0.50 (0.46–0.55) µmol/L respectively. The mean (SD) 3cB_12_ was −0.70 (0.84). The percentile distributions of these bio-markers are presented in Table 2, and the association between these biomarkers and age of infants are depicted in Figure 1. Except for the plasma folate concentrations which were higher (72.5 vs. 62.9 nmol/L, *p* = 0.002) in the fasting samples (no meal or snacks prior to the blood sampling), none of other bio-markers were different according to the history of food/snack consumption before blood sampling. Based on plasma cobalamin concentration <148 pmol/L, only 11% of infants were cobalamin-deficient, whereas 24% had cobalamin concentration between 148–221 pmol/L. None of the infants were found to be folate-deficient when using the conventional cut-off value of folate concentration (<10 nmol/L). The folate status was found to be very good in this population ranging from 20.7 to 150.8 nmol/L. Three-fourths of the infants had elevated MMA levels (>0.28 μmol/L), while 53% had tHcy > 10 μmol/L indicating functional cobalamin deficiency (Table 3). However, based on the 3cB_12_, 58% of the infants had low cobalamin status. The mean Hb was 10.7 g/dL, and anemia was found in 61%, but mostly with a mild degree of severity.

### 3.3. Determinants for Cobalamin Status Biomarkers

Adjusted (and back-transformed when relevant) regression coefficient for 3cB_12_, tHcy, MMA, and cobalamin derived from multiple linear regression analyses are presented in Table 4. Concentrations of tHcy decreased with increasing age (in months) of the infants. Cobalamin concentration and 3cB_12_ increased with age whereas there were no significant association between age and MMA. Infants from a family staying on rent had lower 3cB_12_, and higher MMA and tHcy than those from families staying in their own property. Higher tHcy concentrations were also found among infants who were exclusively breastfed for three months or more. Stunted infants had higher MMA concentrations, whereas underweight infants had higher plasma cobalamin concentrations.

## 4. Discussion

In this cross-sectional analysis consisting of 6–11 months old breastfed infants included over a period of 12 months, we observed cobalamin deficiencies ranging from 11–75% depending upon the indicator that was used. None of the infants had folate deficiency. Based on the combined biomarker (3cB_12_), 58% had low cobalamin status. One in every second infant had high tHcy or MMA suggesting that functional cobalamin deficiency is wide-spread. It should be noted that for cobalamin and the functional markers, there are no well-established cutoff values in infants. Various cutoffs and combinations have been suggested. Bjørke-Monsen et al used a cutoff of 6.5 µmol/L tHcy which was the 95th percentile in infants who had received an injection of vitamin B_12_ [44]. According to this definition, 77% of our infants were functional cobalamin deficient. In a review from 2003, the functional biomarker concentrations from almost 30 studies in young children worldwide were presented [14]. The concentrations of the biomarkers presented in this current study were not very different from those reported from the studies included in this review. Nor were the concentrations from more recent studies in infant populations in Norway [26,45]. In the above-mentioned review and in Norwegian studies, however, none of the infant populations had a mean tHcy > 10 µmol/L while in our study the geometric mean tHcy was 10.8 µmol/L. Equally high tHcy concentrations were observed in other infant and women populations of India and Nepal [6,8]. In these studies, plasma cobalamin and MMA also explained a substantial part of the variability of tHcy. However, the relatively high tHcy compared to MMA concentrations suggests that there are other causes of elevated tHcy (in addition to poor cobalamin status). If this is the case, it will also overestimate the true prevalence when using combined approaches such as those suggested by Fedosov et al. [18] or by Stabler et al. [46].

Our findings of excellent folate status in this population may partly be explained by high iron/folic acid intake by mothers during pregnancy, breastfeeding and the availability of folate in vegetarian foods given to infants [30,47]. Similar findings of high folate status but low cobalamin were found in a recent study from Nepal among 6–23 months old children from Kapilvastu and Achham districts [31], and in our previous studies from the same community among children [8,47] and mothers [7]. In these studies, folate deficiency was also uncommon, but cobalamin deficiency based on low plasma cobalamin ranged from 17–41%. Most of the weaning foods in Nepal are vegetarian/plant-based [48,49] containing fair amounts of folate, particularly in legumes, fruits and nuts. These foods combined with good coverage of folic acid and iron supplementation during pregnancy [30], probably explain the high folate status in this population.

Cobalamin and folate are both essential for erythropoiesis and deficiency may lead to anemia, [50]. Although the prevalence of anemia in our study is quite high, we did not find any association with any of the biomarkers in the regression analyses; a similar observation has previously been made in adult populations [15,51]. Adequate complementary feeding both in terms of quality and quantity is required for infants after six months of age to fulfill their micronutrient requirements [21,32]. In a study in Norwegian infants with a birth weight between 2 to 3 kg, exclusive breastfeeding for at least 3 months was associated with an increased risk of cobalamin deficiency [52]. In our study, children who were exclusively breasted for more than 3 months had significantly higher tHcy concentrations than those who were not. Our finding supports those who argue for maternal B_12_ supplementation, particularly if the mother is vegan and exclusively breastfeeding [53]. However, none of the other vitamin B_12_ indices were associated with exclusive breastfeeding in our study. This could be an indication that plasma tHcy is an unspecific marker of B_12_ status, and that the increase in tHcy also could be due to other causes for example deficiencies in other nutrients, such as vitamin B_2_ or B_6_ [54]. Our findings of associations of house-ownership with 3cB_12_ and tHcy suggests that socio-economic status also plays an important role for cobalamin status among infants. Compared with families who are renting, families having their own house mainly belong to the local Newar ethnic groups, and probably have a better socio-economic status. Another study also found that intake of micronutrients, including cobalamin among infants was associated with higher socio-economic status of the family [55]. An analysis based on the WHO/UNICEF CORE indicators on infant and young child feeding in South Asia found poor scores on minimum dietary diversity, acceptable diet or meal frequency [56]. Although we did not analyze these CORE infant feeding indicators specifically, previous studies from the same community and in Nepal found that intake of micronutrients including cobalamin are often poor [30,57,58].

The main strength of our study is the relatively large community-based sample of breastfed 6–11 months old infants who were screened by clinical examination for acute or chronic illnesses by a physician. Data on cobalamin and folate status covering a panel of biomarkers particularly from healthy infants from this age group are rare. Available published data are based on limited markers in children with a wide age range (6–23 months) [31] or from clinical populations [8,59]. Furthermore, we also collected detailed information on antenatal micronutrient supplementation, which may influence cobalamin and folate status of the offspring [60].

We also recognize some limitations of our study. Due to our enrolment criteria in the main study, our samples are based on infants with length-for-age *z*-scores of <−1, which may limit the generalizability of our findings. This was also reflected in the high prevalence of stunting (33%), in comparison to the 17 to 20% range in this age group in the last demographic survey of Nepal [30]. However, except MMA, none of other indices were associated with stunting. Due to the rapid physiological changes and introduction of different weaning foods, it is also challenging to find acceptable age specific cut-off values for markers of folate and cobalamin during the second half of infancy [14].

## 5. Conclusions

Based on measurement of functional markers of cobalamin status, such as tHcy and MMA and the combined cobalamin indicator (3cB_12_) more than 50% of the infants with length for age <−1 *z*-score may be cobalamin-deficient, which is probably due to sub-optimal maternal nutritional status during pregnancy and lactation or predominantly vegetarian complementary feeding. It is important to identify immediate and long-term consequences of poor cobalamin status during infancy.

## Figures and Tables

**Table 1:** Baseline characteristics of 316 infants participating in a clinical trial on vitamin B12 in Bhaktapur Nepal.

Characteristics	*N* (%)	Mean	SD
Age, months		8.3	1.9
First born child	151 (48)		
Male child	167 (53)		
Home delivery	15 (5)		
Birth weight, gm ^1^		2813	482
Low birth weight (<2500 gm)	52 (17)		
**Demographic features**			
Illiterate or up to grade 5 education of mother	128 (41)		
Illiterate or up to grade 5 education of father	118 (37)		
Not working mother or engaging only in agricultural work	197 (62)		
Mother’s age		27.4	4.7
Family staying in own house	161 (51)		
Family staying in joint family	159 (50)		
Family having own land	145 (46)		
**Breastfeeding status**			
Exclusive breastfeeding for 3 months or more ^2^	137 (43)		
Exclusive breastfeeding for 6 months	33 (10)		
**Nutritional status**			
Underweight (weight for age <−2 *z*-score)	62 (19)		
Stunting (length for age <−2 *z*-score)	106 (33)		
Wasting (weight for length <−2 *z*-score)	13 (6)		
Maternal undernutrition ^3^	34 (6)		

^1^ Among 302 newborns from whom birth weights were available; ^2^ exclusive breastfeeding defined as: the child is given breast milk only, no water or complementary foods, except for medicines. ^3^ BMI < 18.5 kg/m^2^.

**Table 2:** Mean and percentile distributions of cobalamin, folate, hemoglobin, total homocysteine (tHcy), 3cB_12_ and methylmalonic acid(MMA) concentrations among 316 infants participating in a clinical trial on vitamin B_12_ in Bhaktapur Nepal.

Mean/Centile	Cobalamin (pmol/L)	Folate (nmol/L)	tHcy (µmol/L)	MMA (µmol/L)	3cB_12_	Hb (g/dL)
Mean (SD)	304 (152)	65 (23)	12.1 (6.5)	0.74 (0.86)	−0.70 (0.84)	10.7 (0.9)
5%	124	33	5.6	0.15	−2.2	9.4
25%	201	48	7.8	0.27	−1.2	10
50%	272	62	10.3	0.48	−0.64	10.6
75%	364	77	14.2	0.83	−0.13	11.4
95%	601	112	24.8	2.43	0.62	12.3

3cB_12_ = combined indicator of vitamin B_12_ status including 3 biomarkers (cobalamin, MMA, and tHcy), tHcy = total homocysteine, MMA = methylmalonic acid and Hb = hemoglobin concentration.

**Table 3:** Prevalence of cobalamin and folate deficiency and anemia among 316 infants participating in a clinical trial in Bhaktapur, Nepal.

Definition	Cut off	Prevalence (95% CI)
Anemia	<11 gm/dL	61% (55–66)
Altitude adjusted anemia	<11.3 gm/dL	70% (65–75)
Cobalamin deficiency	<148 pmol/L	11% (7–14)
Marginal cobalamin deficiency	148–221 pmol/L	24% (19–29)
Combined indicator of B_12_ (3cB_12_) ^1^	<−0.5	58% (53–64)
Folate deficiency	<10 nmol/L	0
High total homocysteine ^2^ High methylmalonic acid^2^	>10 µmol/L >0.28 µmol/L	53% (47–58) 75% (70–79)

^1^ based on the combined indicator of vitamin B_12_ status including 3 biomarkers (cobalamin, MMA, and tHcy) as suggested by Fedosov et al. [18]. ^2^ both conditions indicate functional B_12_ deficiencies.

**Table 4:** Determinants for the combined cobalamin indicator (3cB_12_) ^1^, total homocysteine (tHcy), methylmalonic acid (MMA), and cobalamin concentrations according to demographic household parameters and nutritional status by multiple linear regression among 316 infants participating in a clinical trial in Bhaktapur, Nepal ^2^.

Independent Variables	Dependent Variables			
	3cB_12_	tHcy	MMA	Cobalamin
Age of child (month)	0.06(0.01, 0.11)	0.93 (0.89, 0.96)	0.99 (0.93, 1.06)	1.05 (1.01, 1.10)
Family not staying in own house	−0.19 (−0.39, 0.01)	1.17 (1.01, 1.36)	1.43 (1.10, 1.85)	
Age of Mother (year)	0.02(0.02, 0.04)	0.98 (0.97, 0.99)		
Father completed education ≤5 year	0.22(0.03,0.41)	0.85 (0.74, 0.98)		
Excl. breastfeeding ≥ 3 months		1.16 (1.01, 1.35)		
Underweight (weight for age <−2 *z*-score)				1.29 (1.07, 1.56)
Stunting (length for age <−2 *z*-score)			1.47 (1.12, 1.93)	

^1^ Combined cobalamin indicator that consists of 3 biomarkers (cobalamin, MMA, and tHcy) [18]. ^2^ Coefficients from by multiple linear regression analysis, MMA, tHcy, and cobalamin were log-transformed in the regression analyses. The regression coefficients and the confidence intervals were back transformed so that the coefficients represent the fold increase in the dependent variable for each unit increase in the independent variable. 3cB_12_ was not log-transformed. For example, the regression coefficient for “Stunting” on MMA is 1.47 which means that the stunted children had a MMA concentration that was on an average 47% higher than the non-stunted children.

## List of Abbreviations

MMA, methylmalonic acid; tHcy- total homocysteine; 3cB_12_-combined indicator of B_12_.
